# Static Calibration and Wiring-Configuration-Dependent Performance of NiCr-Based Thin-Film Thermocouples

**DOI:** 10.3390/mi17060746

**Published:** 2026-06-20

**Authors:** Wenqian Yuan, Zhongfeng Kang

**Affiliations:** School of Information Science and Engineering, Lanzhou University, Lanzhou 730000, China; ywenqian2023@lzu.edu.cn

**Keywords:** thin-film thermocouple, NiCr/NiSi, Seebeck coefficient, static calibration, temperature measurement, uncertainty analysis

## Abstract

Thin-film thermocouples (TFTCs) offer conformal sensing junctions with minimal thermal mass, enabling rapid transient response and direct deposition on curved or moving components, which are difficult to achieve using conventional wire thermocouples in applications such as high-speed machining, electric powertrain thermal management, and fuel-cell monitoring. In practical deployment, the effective accuracy of a TFTC can also be affected by the measurement setup used for calibration and testing, particularly lead-wire material transitions, cold-junction compensation, and wiring-related thermoelectric offsets. This study presents a systematic static calibration and performance evaluation of NiCr-based TFTCs under standardised laboratory conditions, with repeated measurements across the 20–260 °C range using both copper leads and matched compensation wires. The thermoelectric output exhibits excellent linearity; temperature reconstruction against a traceable standard reference yields a maximum deviation of approximately 0.27 °C, with root-mean-square and relative errors within tight bounds. Short-term extended-range verification up to 1000 °C confirms detectable thermoelectric signal generation under the present test conditions. A calibration data packet framework containing the calibrated TFTC sample, wiring configuration, calibration coefficients, validity range, and a GUM-compliant uncertainty budget is proposed to support consistent interpretation of calibration results in future digital integration. The study therefore provides a structured calibration workflow and uncertainty-reporting basis for the tested flexible NiCr-based TFTC configurations, supporting further reliability assessment, material-level characterisation, and digital integration.

## 1. Introduction

Accurate local temperature information is important for understanding and controlling systems where thermodynamic processes strongly influence performance, safety, and operational longevity [1]. Representative applications include high-speed machining, where local cutting-zone temperatures affect tool wear [2]; electric powertrains, whose efficiency depends on thermal state [3,4,5]; proton exchange membrane fuel cells, where temperature distribution influences water management [6]; and biomedical drilling, in which thermal excursion must be contained to prevent tissue necrosis. In these scenarios, temperature varies rapidly in both time and space, and the physically meaningful signal often resides in local surface temperature rather than bulk averages [7].

Conventional wire thermocouples, though durable and industrially standardised, can be limited in such applications. Their junction mass and thermal inertia may attenuate fast transients, routing wires onto moving or compact components can be difficult, and measurement points may be thermally remote from actual hot spots [8]. Thin-film thermocouples, fabricated by micro- and nano-scale deposition techniques, form small sensing junctions directly on target surfaces [9], enabling array-based temperature-field mapping [7] and integration with curved or constrained surfaces [10]. While operating on the same Seebeck effect as wire thermocouples, TFTCs experience distinct thermal and electrical boundary conditions. The effective Seebeck coefficient can be sensitive to film-specific factors [11], and the substrate and protective layers participate in the thermal response of the sensing structure [12].

Related industrial measurement research also shows that no single diagnostic method is sufficient for complex thermal-fluid systems. Yan et al. reviewed experimental techniques for industrial-scale multiphase-flow problems, including laser Doppler velocimetry, particle image velocimetry, and particle/droplet image analysis, and emphasised that measurement reliability depends on selecting techniques appropriate to the physical process and scale of interest [13]. Li et al. further showed, using a supercritical-water flat-plate model, that strong property variations in boundary layers require careful treatment of the physical model and measurement interpretation [14]. These studies highlight a broader point relevant to TFTC deployment: sensor outputs in complex thermal systems must be interpreted together with the measurement configuration and its uncertainty.

Recent studies have reported important improvements in TFTC materials, calibration, and application-specific performance. Liu et al. systematically characterised the NiCr/NiSi thin-film Seebeck coefficient as a function of film thickness, reporting values from 7.3 to 21.3 μV/°C and identifying the calibration procedure itself as a decisive factor in determining measured sensitivity [11]. Cui et al. reported a NiCr/NiSi TFTC with a Seebeck coefficient of 40.72 μV/°C and a 0.70 ms dynamic response for milling temperature monitoring [15]. Lian et al. achieved 48.13 μV/°C on a cutting tool insert and validated durability over 1500 m of cutting length [2]. Wang et al. used 7.6 ns short-pulse laser excitation to reveal second-order dynamic behaviour in NiCr/NiSi TFTCs [16], and Sun et al. independently reported thermoelectric oscillation phenomena under pulsed laser excitation [17]. Beyond the NiCr/NiSi system, Ma et al. achieved calibration repeatability better than 0.21% deviation over 300–1000 °C with highly oriented Pt/Ir films [18]. Wang et al. reported Pt-Rh10/Pt screen-printed TFTCs with a repeatability error of 0.08% over 50–1500 °C [8,19], and Xie et al. demonstrated ITO/In_2_O_3_ TFTCs with a Seebeck coefficient of 129.3 μV/°C over 200–1450 °C [20].

These studies report important progress in TFTC materials, fabrication, calibration, and application; however, selected measurement-setup factors, especially wiring topology and reference conditions, are still often treated as external acquisition issues rather than as part of the calibration definition. This distinction matters because wiring materials, pad-to-lead transitions, connectors, cold-junction compensation, and terminal temperature gradients can introduce parasitic thermoelectric voltages [11,21].

These considerations have practical and metrological implications. Parasitic thermoelectric voltages, cold-junction compensation uncertainty, and connector temperature gradients can introduce systematic bias if they are not considered during calibration and deployment. For digital monitoring or multi-sensor fusion, the calibration record must also specify the conditions under which the calibration coefficients remain valid [22,23]. Yang demonstrated the value of integrated calibration-coefficient treatment for thin-film heat flux gauges, achieving a relative expanded uncertainty below 7% [24]. For NiCr-based TFTCs, a comparable need exists to record the wiring configuration, reference condition, calibration coefficients, validity range, and uncertainty budget together with the calibrated device.

Accordingly, the present study examines the calibration behaviour of NiCr-based TFTCs under clearly defined wiring configurations, with particular attention to the wiring condition, reference condition, calibration model, and uncertainty budget. Two NiCr-based TFTC variants with different electrode pairs were calibrated under both copper–lead and compensation-wire configurations, with five independent repetitions per condition to evaluate run-to-run repeatability. Temperature reconstruction accuracy was assessed against a traceable standard reference, and dynamic behaviour was characterised under the adopted transient-excitation protocol. The main contribution is a wiring-configuration-aware calibration workflow and a calibration data packet that records the calibrated TFTC sample, wiring configuration, calibration coefficients, validity range, and GUM-compliant uncertainty budget. This packet is intended to support consistent interpretation of calibration results within the tested configuration. Accordingly, the digital twin discussion in this work is limited to calibration-data structuring and future integration rather than a completed system-level implementation.

The present work is therefore positioned as a calibration-focused study of two independently designed NiCr-based TFTC samples under defined laboratory conditions. The dataset supports comparison of the tested configurations in terms of effective sensitivity, temperature reconstruction, wiring-configuration dependence, short-term high-temperature signal generation, and apparent system-level transient response. Batch-level manufacturing reproducibility, long-term high-temperature durability, intrinsic junction bandwidth, and direct microstructure–property correlation require dedicated follow-up experiments. Thus, the high-temperature and transient-response results are interpreted within the tested conditions and are not extended to lifetime reliability or intrinsic junction-bandwidth claims.

## 2. Thermoelectric Principles and Device Design of Thin-Film Thermocouples

### 2.1. Thermoelectric Principle and Operating Mechanism of TFTCs

The thermoelectric potential, *E*, generated between the sensing junction and the reference junction of two dissimilar thermoelectric legs A and B can be expressed as (1):

(1) $$E=\int_{T_{ref}}^{T_{hot}}\left[S_{A}\left(T\right)-S_{B}\left(T\right)\right]dT,$$

where *T_hot_* and *T_ref_* are the sensing-junction and reference-junction temperatures, respectively, and *S_A_(T)* and *S_B_(T)* are the temperature-dependent Seebeck coefficients of the two thermoelectric legs. Over the 20–260 °C calibration range used in the primary static tests, the difference between the two Seebeck coefficients can be approximated as nearly constant [11]. The thermoelectric potential can therefore be written as (2):

(2) $$E=S_{eff}\cdot \Delta T=S_{eff}\cdot \left(T_{hot}-T_{ref}\right),$$

where *S_eff_* is the effective Seebeck coefficient of the thermocouple material pair [12]. In this work, the calibration procedure regresses E against the reference temperature under a specified wiring configuration, and *S_eff_* is determined from the regression slope. The obtained coefficient represents a sensor-level effective coefficient of the tested TFTC calibration configuration under a specified wiring condition, rather than the intrinsic material constant of an individual thin-film. For operation over a wider temperature range, higher-order thermocouple reference functions may be required; unless otherwise validated, the first-order model used here is limited to the calibrated temperature range.

In TFTCs, the thermoelectric legs are deposited and patterned as thin films on a substrate rather than used as free-standing wires [1,10]. This architecture can provide low thermal mass for rapid response [16,17], high spatial resolution for local and array-based surface-temperature measurement [7,25], and conformal integration on curved or constrained surfaces [10,26]. These advantages are also coupled with film-specific and system-level effects, including sensitivity to film microstructure and oxidation [11,27,28], the need for robust insulation and protection [29], and transient heat transfer through the substrate and protective layers [16,27]. These TFTC-specific factors motivate the measurement-chain-aware calibration strategy used in this work.

### 2.2. Material Selection and Layer Stack Design of TFTCs

Thermoelectric materials for temperature sensing and those for thermoelectric energy conversion are selected by different criteria. Bi_2_Te_3_-based compounds are representative high-performance materials for near-room-temperature energy conversion and cooling [30]. However, thermocouples are designed to generate a stable and repeatable thermoelectric potential under a temperature difference, rather than to maximise power conversion. Therefore, TFTC material selection should also consider oxidation resistance, diffusion stability, thin-film deposition compatibility, adhesion to insulating layers, and the intended operating temperature range. Representative thermocouple material systems include Ni-based type-K-equivalent alloys, noble metal pairs such as Pt/PtRh and Pt/Ir, and high-temperature oxide semiconductor pairs such as ITO/In_2_O_3_. Ni-based systems offer a practical balance of sensitivity, cost, and sputtering compatibility. Noble metal systems provide better high-temperature stability at a higher cost. Oxide semiconductor systems may provide high sensitivity at an elevated temperature, but their film stoichiometry and stability require careful control.

NiCr (chromel-like) and NiSi (nisil/alumel-like) constitute the principal material pair for type-K-equivalent TFTC fabrication [1,6]. Ni-based alloys were selected because their effective Seebeck response can approach the nominal value of standard type-K thermocouples after proper calibration [11,12]. They are also compatible with magnetron sputtering [31] and are less costly than noble metal thermoelectric materials. The electrode material choice is closely linked to the insulating, protective, and adhesion-related layers, because layer material, thickness, and interface quality affect thermoelectric output, reliability, and measurement accuracy [27,29,32]. The NiCr/NiSi sample is the principal type-K-equivalent TFTC investigated in this work. A second NiCr/Au sample was included as a comparative NiCr-based structure, rather than as a replacement for the NiCr/NiSi system. Au was selected because of its high electrical conductivity and chemical stability within the tested temperature range. This makes it suitable for flexible sensing configurations where low lead resistance and high signal-to-noise ratio are desirable. Its inclusion allows the measurement-chain-aware calibration strategy to be examined on a second NiCr-based material pair and helps compare device-specific sensitivity with wiring-configuration-dependent effects.

Two differentiated samples were designed. Sample 1 employs a NiCr/Au thermoelectric pair in a stacked configuration. The layer sequence is NiCr → ZnO → Au → SiO_2_. ZnO serves as the interlayer dielectric between the thermoelectric films, and SiO_2_ serves as the protective overcoat. The PI substrate thickness is 0.05 mm. The NiCr layer, Au counter-electrode, ZnO dielectric layer, and SiO_2_ protective layer are 200 nm, 300 nm, 500 nm, and 200 nm thick, respectively. The rectangular hot junction is 5.5 ± 0.5 mm × 5.5 ± 0.5 mm, and the overall substrate dimensions are 60.0 ± 0.5 mm × 60.0 ± 0.5 mm. This configuration targets flexible, high-signal-to-noise-ratio sensing applications. Sample 2 employs a NiCr/NiSi thermoelectric pair in a planar configuration. The layer sequence is Al_2_O_3_ → NiCr → NiSi → SiO_2_. Al_2_O_3_ serves as the base dielectric layer beneath the thermoelectric films, and SiO_2_ serves as the protective overcoat. The PI substrate thickness is 0.05 mm. The NiCr and NiSi layers are 200 nm and 300 nm thick, respectively. The Al_2_O_3_ dielectric layer and SiO_2_ protective layer are 600 nm and 200 nm thick, respectively. The rectangular hot junction is 6.8 ± 0.5 mm × 2.9 ± 0.5 mm, and the overall device dimensions are 10.0 ± 0.5 mm × 6.8 ± 0.5 mm. This configuration is intended for industrial high-temperature cutting-temperature measurement.

No additional metallic adhesion layer was introduced in either sample. Instead, surface activation pretreatment of the PI substrate was used to enhance interfacial bonding with the inorganic insulation layer. This helps avoid the internal stress that a metallic adhesion layer could introduce and helps preserve substrate flexibility [10,29]. Full fabrication details, including deposition parameters, are provided in Section 3.1. The practical performance trade-offs among representative thermoelectric and electrode materials for thin-film temperature sensing are summarised in Table 1.

### 2.3. Device Packaging, Wiring Configuration and Cold-Junction Compensation (CJC)

While the hot junction is the primary sensing element, the wiring configuration determines whether the measured voltage corresponds to the intended thermocouple junction. In an ideal thermocouple circuit, only the hot and reference junctions contribute. In real systems, additional junctions are introduced at connectors, solder joints, and transitions from thermoelectric materials to copper wiring. Each dissimilar-metal junction can generate a parasitic thermoelectric voltage if it experiences a temperature gradient [12,21]. Therefore, wiring and connector design are fundamental to measurement accuracy.

Cold-junction compensation (CJC) is needed because the thermocouple voltage corresponds to a temperature difference [1,3]. Commercial instruments typically measure the terminal temperature and add an equivalent compensation voltage. However, in a thin-film thermocouple system, the effective reference junction may not coincide with the instrument terminals if intermediate material transitions are present. The effective reference temperature must therefore be defined carefully, and intermediate junctions should be kept isothermal through thermal anchoring when possible.

In this work, two wiring configurations were systematically compared: direct copper leads attached to the TFTC electrode pads and matched type-K compensation wires connecting the TFTC pads to the data acquisition system. The conceptual role of wiring and CJC is described in this section, whereas the experimental implementation of the two wiring configurations is described in Section 3.2.1. The comparison focuses on the influence of wiring configuration on the effective Seebeck coefficient and measurement repeatability. This comparison is based on two independently designed TFTC samples and supports a preliminary assessment of measurement repeatability; verification of batch-level consistency would require a larger sample cohort.

## 3. Experimental Methods

### 3.1. Device Fabrication and Layer-Stack Architecture

Two TFTC samples with application-specific designs were independently fabricated on flexible polyimide (PI) substrates [1,10]. The material pairs, layer sequences, nominal layer thicknesses, hot-junction dimensions, and overall device dimensions are defined in Section 2.2. To keep the comparison controlled, the thermoelectric material pairing, hot-junction geometry, and dielectric-layer material were varied between the two samples, while the remaining fabrication process, deposition conditions, and environmental conditions were kept consistent.

**Figure 1:**
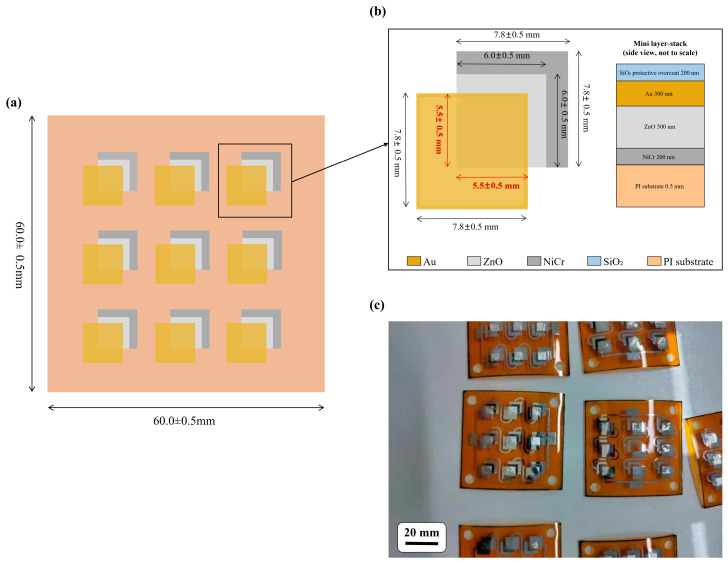
Schematic diagrams and optical photograph of the as-fabricated NiCr/Au TFTC array on a PI flexible substrate. (**a**) Overall layout of the 3 × 3 TFTC array. (**b**) Stacked structure and dimensional parameters of a single TFTC sensing unit, with clearer material labels for NiCr, Au, ZnO, the PI substrate, and the SiO_2_ protective layer. (**c**) Optical photograph of the as-fabricated flexible TFTC array samples, with a dimension-referenced scale bar based on the reported overall substrate dimension.

The dielectric, thermoelectric, and protective layers were deposited by dual-chamber magnetron sputtering using argon as the working gas. The working pressure was 0.7 Pa, the target-to-substrate distance was 120 mm, and the sputtering power density was 1.9 W/cm^2^. All targets had a purity of 99.99%; the NiCr and NiSi target compositions were 80:20 wt.% and 95:5 wt.%, respectively. The substrate was maintained at room temperature without auxiliary heating, and the sample stage was continuously rotated to enhance film uniformity across the substrate surface [33]. No metallic adhesion layer was introduced, and no post-deposition annealing was performed. A SiO_2_ protective overcoat was deposited to mitigate oxidation and environmental degradation during testing [33]. Detailed procedural information, including substrate pretreatment, patterning implementation, electrode-pad opening, wire bonding, curing, and lead protection, is provided in Supplementary Table S1. The NiCr/Au TFTC array fabricated on the PI flexible substrate is shown in Figure 1.

**Figure 2:**
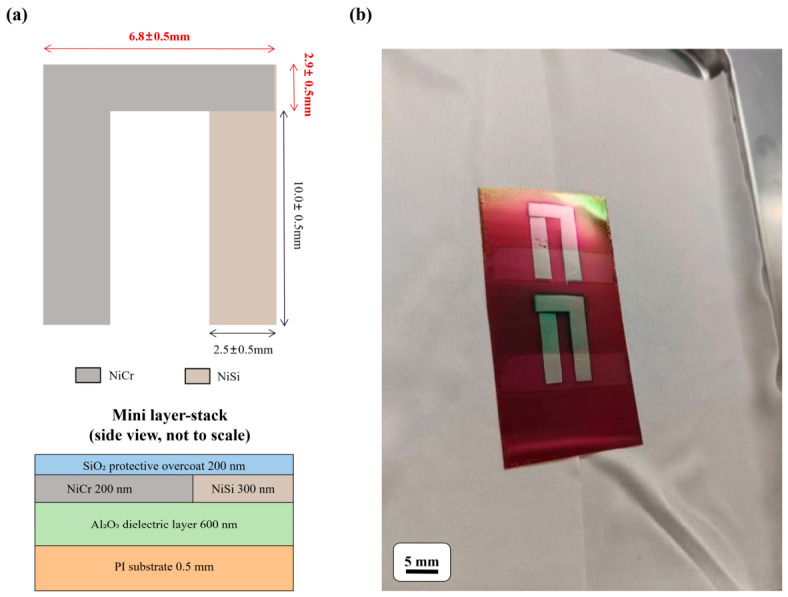
Schematic layout and optical photograph of the as-fabricated NiCr/NiSi TFTC sensing junction. (**a**) Planar structure and dimensional parameters of the TFTC sensing junction, with clearer material labels for NiCr, NiSi, the PI substrate, the Al_2_O_3_ dielectric layer, and the SiO_2_ protective layer. (**b**) Optical photograph of the as-fabricated NiCr/NiSi TFTC sample, with a dimension-referenced scale bar based on the reported overall device dimension.

The same fabrication route was used for the NiCr/NiSi TFTC, with the material pair, dielectric-layer material, and hot-junction geometry adjusted according to the design defined in Section 2.2. The fabricated device adopts a planar butt-junction structure, as shown in Figure 2.

The characterisation reported in this section is limited to device geometry, process-defined layer information, deposition conditions, and optical inspection of the fabricated samples. Detailed crystallographic, compositional, residual-stress, interfacial-diffusion, and high-temperature microstructural evolution analyses are outside the experimental scope of the present calibration-focused study. Accordingly, the calibration results are interpreted at the device and wiring-configuration level, rather than being used to claim a quantitative causal relationship between microstructure and sensing performance.

### 3.2. Test System and Measurement Configuration

#### 3.2.1. Wiring Configurations Under Test

Two wiring configurations were compared to quantify the influence of wiring transitions on the effective Seebeck coefficient and temperature reconstruction accuracy. The configurations were defined as follows and were used consistently throughout the calibration and response tests. In Configuration A (copper-lead), the TFTC electrode pads were directly connected to 0.6 mm-diameter copper wires via high-temperature conductive silver paste, with the copper wires leading directly to the data acquisition instrument. This configuration is straightforward to implement but introduces parasitic thermoelectric junctions at the pad–wire transition and at the instrument terminals [21]. In Configuration B (compensation wire), matched type-K compensation wires were connected between the TFTC pads and the data acquisition instrument. This configuration reduces parasitic thermoelectric contributions from dissimilar-material transitions along the signal path. For both configurations, all accessible electrical connection joints were maintained under near-isothermal conditions through thermal anchoring to minimise additional parasitic effects [3]. The same calibration protocol, reference thermocouple, furnace conditions, and data-processing procedure were used for both configurations so that the observed differences could be attributed primarily to the wiring topology.

#### 3.2.2. Core Measurement Instruments

Static calibration thermoelectric voltages were acquired using a commercial thermocouple input module, with a measurement accuracy of ±0.50 °C, cold-junction compensation accuracy of ±0.50 °C, and voltage resolution of 0.01 mV. A calibrated type-K standard reference thermocouple with an accuracy of ±0.05 °C over 20–260 °C served as the temperature reference. Stable and uniform temperature fields were provided by low-temperature and high-temperature verification furnaces with a temperature control accuracy of ±0.10 °C and an operating range of 50–1500 °C. The primary calibration range was 20–260 °C, with extended verification up to 1000 °C. For dynamic response testing, transient signals were recorded using a commercial memory hi-corder/data acquisition recorder with a sampling frequency of 10–100 kHz, low-pass filtering enabled, and full synchronisation with the laser trigger. Step-temperature excitation was generated by a high-speed pulsed infrared laser [33].

### 3.3. Experimental Test Protocol

#### 3.3.1. Static Calibration Protocol

Static calibration was performed under both wiring configurations defined in Section 3.2.1 over 20–260 °C at nine standardised setpoints (20, 50, 80, 110, 140, 170, 200, 230, and 260 °C). The TFTC samples and the standard reference thermocouple were co-located in the uniform temperature zone, with the reference junction maintained at a stable room temperature through thermal anchoring. At each setpoint, the system was held for 30 min to ensure complete thermal equilibrium prior to data acquisition [3]. For each sample and wiring configuration, five independent repeated calibration runs were performed. Extended verification up to 1000 °C was conducted for Sample 2 to evaluate high-temperature applicability. This study is based on two independently designed TFTC samples, which supports a preliminary assessment of measurement repeatability; batch consistency verification requires a larger cohort.

#### 3.3.2. Dynamic Response Test Protocol

Dynamic response was characterised in ambient air using a step-change heating test, with the initial temperature stabilised at room temperature (20–25 °C). The step-change thermal excitation was produced by a pulsed infrared heat source with a single-pulse energy of 15.49–29.59 mJ, a nominal pulse width of 7.6 ns, and a spot diameter of 2.56 mm [16,17]. The beam was directed toward the hot-junction region of the TFTC to produce a rapid temperature change at the sensing area. The excitation start time was defined as t = 0, and the excitation trigger was synchronised with the data acquisition system. The transient thermoelectric potential *E* was then recorded using a high-speed dynamic signal acquisition and oscilloscope recording system. A two-channel acquisition configuration was used, with a sampling frequency in the 10–100 kHz range and low-pass filtering enabled. Three repeated tests were performed for each sample, and the averaged waveform was used for subsequent analysis. The extracted dynamic metrics include the 10–90% rise time, time-to-peak, and settling time. The wiring definitions used for this test are those specified in Section 3.2.1.

### 3.4. Data Processing, Statistical Analysis and Uncertainty Evaluation

#### 3.4.1. Calibration Model and Temperature Reconstruction

For each calibration run, the measured thermoelectric voltage *E* was regressed against the reference temperature *T* from the calibrated standard thermocouple using a first-order linear model over the 20–260 °C range (3), consistent with industrial practice for type-K thermocouples in this temperature interval [11,12]:

(3) $$E=a\cdot (T-T_{0})+b,$$

where *E* is the thermoelectric potential, *a* is the effective Seebeck coefficient (μV/°C), *b* is the offset voltage at the reference temperature, and *T*_0_ is the reference calibration temperature used to reduce parameter correlation. Slope a was taken as the effective Seebeck coefficient of the TFTC over the tested temperature range. Temperature reconstruction was performed using the inverse linear model (4):

(4) $$T=\frac{E-a}{b}+T_{0},$$

where *T* is the temperature reconstructed from the measured thermoelectric potential *E*.

#### 3.4.2. Error Quantification and Statistical Analysis

Temperature measurement error was quantified using three metrics, with the reference temperature from the calibrated standard thermocouple taken as the true value [12]. The pointwise absolute error (5) was defined as:

(5) $$E_{i}={\hat{y}}_{i}-y_{i},$$

where *y_i_* is the reference temperature at setpoint *i* and *ŷ_i_* is the TFTC-reconstructed temperature. The maximum absolute error (6) was taken as:

(6) $$E_{i}=\frac{\left({\hat{y}}_{i}-y_{i}\right)}{y_{i}}\times 100\%,$$

The maximum absolute error (7) was taken as:

(7) $$L_{\infty }=\underset{1\leq i\leq n}{\max}|{\bar{y}}_{i}-y_{i}|,$$

and the root-mean-square error (8) was calculated as:

(8) $$RMSE=\sqrt{\frac{1}{n}\sum_{i=1}^{n}({\bar{y}}_{i}-y_{i}{)}^{2}},$$

Measurement repeatability was quantified by the coefficient of variation in the effective Seebeck coefficient across the five repeated calibration runs for each sample and configuration. The slope estimate from multi-point regression is less sensitive to single-point noise than individual voltage readings and provides a stable summary of device sensitivity; the distribution of slopes across runs captures practical variability from contact repeatability, thermal equilibrium, and instrument drift [12]. Sample-to-sample variability was evaluated by comparing the effective Seebeck coefficients of the two samples under identical test protocols.

#### 3.4.3. Measurement Uncertainty Evaluation

Measurement uncertainty was evaluated in accordance with the Guide to the Expression of Uncertainty in Measurement (GUM). The uncertainty budget combines Type A uncertainty, derived from the standard deviation of the measured Seebeck coefficient across repeated calibration runs, and Type B uncertainty, derived from the reference thermocouple, cold-junction compensation accuracy, voltage measurement resolution, and linear regression model residuals. The combined standard uncertainty was calculated via the root-sum-square method, and the expanded uncertainty U was determined with a coverage factor k = 2 (95% confidence level). 

## 4. Results and Discussion

### 4.1. Static Calibration Curves and Linearity

#### 4.1.1. Room-Temperature to 260 °C Calibration and Linearity

**Figure 3:**
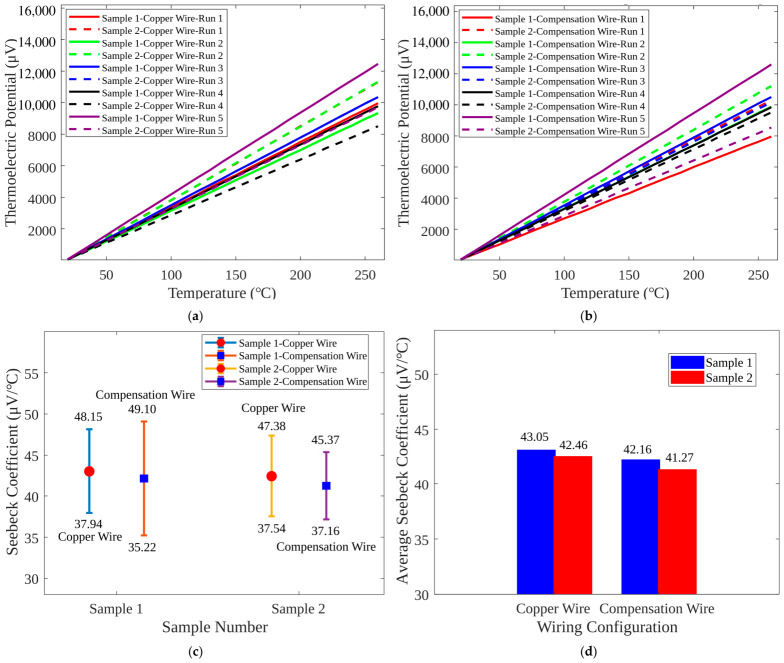
Static calibration and effective Seebeck coefficient analysis of NiCr-based TFTCs under different wiring configurations (20–260 °C, *n* = 5). (**a**) Thermoelectric potential *E*–temperature curves with copper leads; (**b**) thermoelectric potential *E*–temperature curves with compensation wires; (**c**) min–max range of the effective Seebeck coefficient across five repeated runs; (**d**) comparison of the mean effective Seebeck coefficient between copper-lead and compensation-wire configurations.

Static calibration was conducted over 20–260 °C with five independent repeated runs for each sample and wiring configuration. The thermoelectric potential *E*–temperature characteristics and the corresponding effective Seebeck coefficient analysis are presented in Figure 3. The five repeated traces for each condition (Figure 3a,b) are nearly coincident, yielding a coefficient of variation below 0.3% across all test groups and confirming excellent run-to-run repeatability. Thermoelectric potential E increases linearly with temperature over the full range, consistent with the expectation that the Seebeck coefficient of Ni-based thermoelectric pairs remains approximately constant in this moderate-temperature interval [11,12]. Comparable linearity has been reported for NiCr/NiSi TFTCs on polyimide substrates over 20–350 °C [3] and on alumina ceramic substrates for milling temperature monitoring [15], indicating that linear thermoelectric output is a reproducible characteristic of this material system under properly controlled fabrication and calibration protocols. The min–max range of the effective Seebeck coefficient across repeated runs and the mean values for the two samples under each wiring configuration are shown in Figure 3c,d. The raw calibration data and corresponding linearity metrics are summarised in Table 2.

#### 4.1.2. Effect of Wiring Configuration on Measurement Sensitivity

Table 3 summarises the effective Seebeck coefficient statistics derived from linear regression for each sample and wiring configuration (*n* = 5 per condition). To avoid ambiguity in interpreting Figure 3c, and Table 3, the Seebeck coefficient spread is separated into run-to-run repeatability, wiring-configuration dependence, and sample-to-sample design variation. The min–max interval shown in Figure 3c represents the distribution of fitted slopes obtained from repeated calibration runs; it should not be interpreted as a direct temperature-measurement error range. Between the copper-lead and compensation-wire configurations, the mean sensitivity differs by ~0.4 μV/°C for Sample 1 and ~0.44 μV/°C for Sample 2, corresponding to a relative difference of approximately 1%. Although this difference is small in relative terms, it may produce a measurable temperature bias over wide temperature spans if the wiring configuration is changed without recalibration. This difference is consistent with contributions from parasitic thermoelectric potentials generated at dissimilar-metal junctions formed when copper leads interface with the thin-film electrode pads; these parasitic contributions superimpose on the target TFTC signal and modify the effective voltage–temperature slope [21]. Matched type-K compensation wires can reduce this contribution by better matching the thermoelectric properties of the NiCr–NiSi signal path under near-isothermal connection conditions. The larger difference between Sample 1 and Sample 2 reflects their intentionally different material pairs, dielectric stacks, and hot-junction geometries, rather than instability of a single device. Consistent repeatability within each fixed sample and wiring configuration (CV < 0.3%) suggests that the tested configuration is repeatable once the wiring topology is defined. In this work, performance consistency is therefore interpreted primarily from within-configuration repeatability and temperature-reconstruction error, whereas inter-sample and inter-wiring differences are treated as calibration-definition effects that should be recorded in the calibration data packet. Prior calibration studies on Pt-Rh10/Pt [8] and ITO/In_2_O_3_ [20] TFTCs have reported Seebeck coefficients and repeatability metrics without explicitly isolating the contribution of wiring configuration. For industrial deployment, the wiring configuration should therefore be recorded as part of the sensor calibration definition: a TFTC calibrated with copper leads should not be used with compensation wires unless recalibration or a validated correction model is applied.

#### 4.1.3. Extended High-Temperature Calibration up to 1000 °C

**Figure 4:**
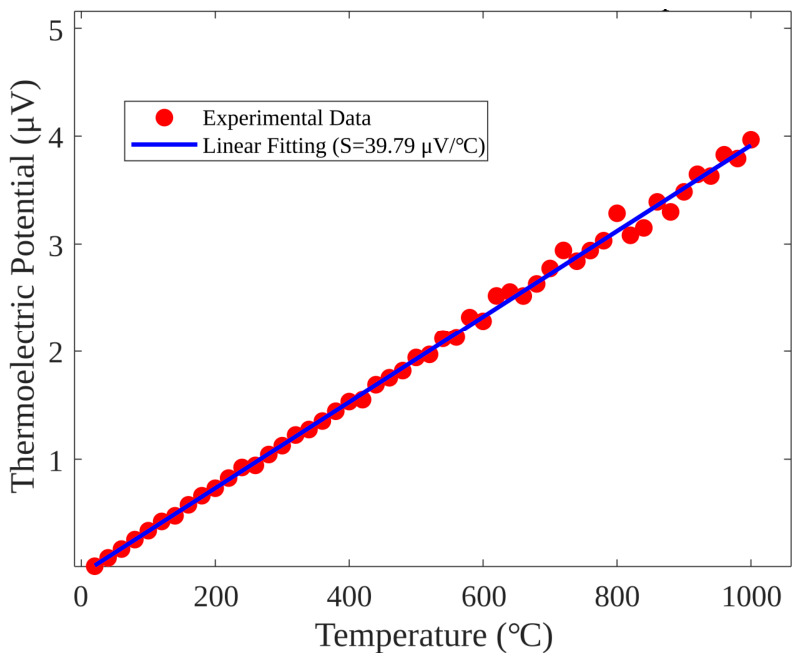
Extended-range static calibration curve of NiCr/NiSi TFTC up to 1000 °C with linear fitting.

Extended static calibration was performed up to 1000 °C to examine the short-term high-temperature calibration response of Sample 2 under the present laboratory conditions. The calibration curve and linear fitting results are shown in Figure 4. The thermoelectric potential *E* reaches approximately 40 mV at 1000 °C, with a fitted effective Seebeck coefficient of 39.79 μV/°C and a correlation coefficient R^2^ > 0.99. This result indicates that the NiCr/NiSi TFTC produces a measurable thermoelectric signal over the extended test range, and it is interpreted here as short-term extended-range verification rather than long-term durability validation. The slight reduction in sensitivity relative to the 20–260 °C range may be associated with the temperature dependence of NiCr/NiSi thermoelectric properties [11,32] and possible thermal changes in the sputtered thin films during high-temperature exposure. Because post-test microstructural characterisation was not included in this study, this trend is discussed as a calibration observation and is not assigned to a specific microstructural mechanism.

For context, Ma et al. reported that highly oriented Pt/Ir thin-film thermocouples maintained a calibration deviation below 0.21% over three cycles up to 1000 °C [18], and Xie et al. demonstrated ITO/In_2_O_3_ TFTCs with a Seebeck coefficient of 129.3 μV/°C over 200–1450 °C [20]. Ruan et al. reported rapid thermoelectric failure of NiCr/NiSi film thermocouples on silicon when the hot junction reached 600 °C, highlighting the importance of substrate and protective-layer selection in high-temperature survivability [32]. The present result supports short-term high-temperature signal verification for the tested NiCr/NiSi configuration. Long-term drift, oxidation resistance, interface stability, and repeated high-temperature cycling remain reliability issues to be evaluated under application-relevant thermal, mechanical, and environmental conditions.

### 4.2. Measurement Accuracy, Reliability and Dynamic Response Characteristics

#### 4.2.1. Temperature Reconstruction Accuracy and Error Analysis

**Figure 5:**
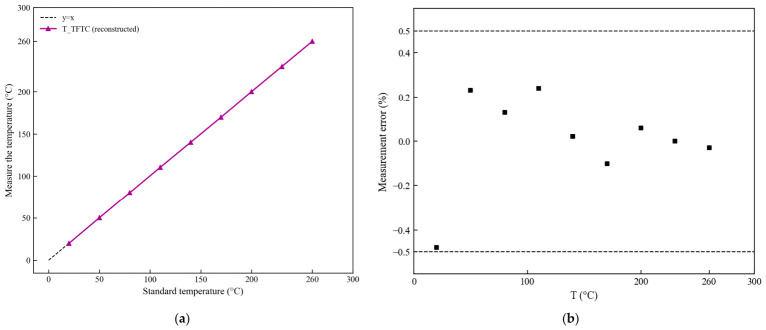
Temperature measurement performance evaluation of the NiCr/NiSi TFTC measurement chain (20–260 °C): (**a**) comparison between temperature reconstructed from the NiCr/NiSi TFTC and that from a standard K-type reference thermocouple; (**b**) distribution of percentage error in the TFTC temperature measurement (the black squares denote the percentage errors at the nine standard temperature points defined in Section 3.3.1, and the dashed lines indicate the ±0.5% error bounds.).

Temperature reconstruction was performed using the inverse linear calibration model established in Section 3.4.1, with a calibrated standard type-K thermocouple serving as the reference. The reconstructed temperature versus the standard setpoint and the relative error distribution are shown in Figure 5. Over the full 20–260 °C range, the TFTC measurement chain achieves a maximum absolute error of ≤0.27 °C and a root-mean-square error (RMSE) of 0.15 °C. The relative error remains within ±0.5% for all setpoints, with the maximum value of −0.48% occurring at 20 °C, where inherently small absolute deviations translate into larger relative percentages; at all other setpoints the relative error is within ±0.25%.

#### 4.2.2. Long-Term Repeatability and Thermal Cycle Stability

To provide a preliminary check of repeatability under repeated heating and cooling, 20 thermal cycles (20 °C → 100 °C → 20 °C) were performed, with the steady-state temperature recorded at the fixed calibration point of 100 °C for each cycle. The mean measured temperature across all cycles is 99.9987 °C, with a coefficient of variation of 0.2077%. The maximum absolute deviation from the initial calibrated value remains below 0.5 °C across the 20 cycles. Representative results at key cycle nodes are summarised in Table 4. This result indicates repeatable output under the limited thermal-cycle protocol used here.

This test is described as a limited thermal-cycle repeatability assessment. It should not be interpreted as a long-term drift or durability evaluation under sustained high-temperature exposure or complex industrial loading. Direct quantitative comparison with the inter-cycle deviation reported for Pt/Ir thin-film thermocouples [18] and the performance reported for Pt-Rh10/Pt screen-printed TFTCs [8] remains constrained by differences in material systems, temperature ranges, and cycling protocols.

#### 4.2.3. Transient Dynamic Response and Bandwidth Characteristics

**Figure 6:**
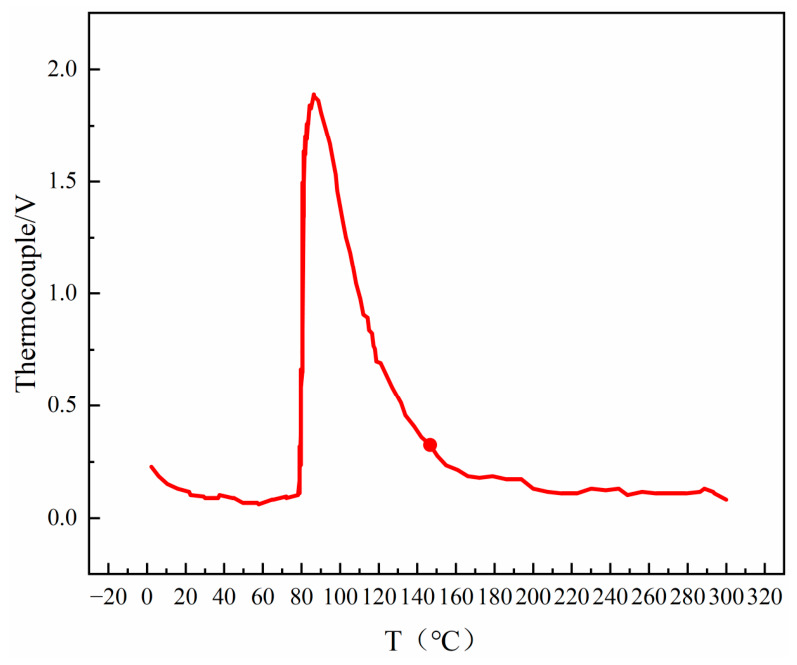
Dynamic response waveform of the TFTC under a step temperature excitation (the red dot indicates a representative data point used for transient response evaluation).

The transient dynamic response was characterised using a step-change heating test, with the averaged waveform from three repeated tests shown in Figure 6. The waveform shows a smooth rise in thermoelectric output without obvious overshoot or oscillation, followed by gradual stabilisation. Under the adopted excitation protocol, the tested TFTC configuration exhibits a 10–90% rise time of approximately 56 ms, a time-to-peak of approximately 95 ms, and a settling time of 120.0 ms, where the settling time is defined as stabilisation within ±2% of the steady-state amplitude. The relatively fast response is consistent with the reduced thermal mass of the thin-film thermoelectric junction and the close thermal coupling between the sensing junction and the substrate [16,17]. Compared with conventional armoured type-K wire thermocouples tested under the same conditions, the TFTC shows a faster transient temperature response, supporting its suitability for millisecond-scale temperature-change monitoring. The post-peak decay and stabilisation behaviour are mainly associated with heat spreading into the substrate and heat dissipation to the surrounding environment [27]. These results demonstrate that the fabricated TFTC can respond rapidly and reproducibly to step-change thermal excitation under the present test conditions.

### 4.3. Comparison with Representative Thermocouples and TFTC Studies

The as-fabricated TFTC exhibits an effective Seebeck coefficient (40.09–41.65 μV/°C) comparable to that of standard type-K wire thermocouples (~41.2 μV/°C) type-J thermocouples offer higher sensitivity (~55.0 μV/°C) but are generally limited to service below 800 °C. At 1000 °C, the TFTC Seebeck coefficient (39.79 μV/°C) remains within the expected range for type-K devices, consistent with ASTM E230 specifications. The 10–90% rise time (~56 ms) is substantially faster than that of conventional armoured wire thermocouples, which typically exceed 100 ms under identical test conditions [3,16]; wire thermocouples are therefore better suited to static and quasi-static measurements [17]. Thermal drift mechanisms differ between the two technologies: oxidation and interfacial interdiffusion dominate in thin-film devices, whereas elemental diffusion within the alloy bulk governs wire-thermocouple drift [32]. Conventional wire thermocouples retain advantages in long-term calibration stability and low-cost large-scale deployment [11]. These comparisons are summarised in Table 5.

Across the recent NiCr/NiSi TFTC literature, reported Seebeck coefficients span 34.96–48.13 μV/°C depending on substrate and fabrication route [2,3,15,25,34]; the present values fall within this range. Notably, explicit quantification of wiring-configuration effects and inclusion of a GUM-compliant uncertainty budget—both addressed in this work—are absent from the studies included in this comparison, which report sensitivity and repeatability without isolating the measurement-chain contribution [8,18,20,34]. A structured comparison of representative TFTC studies is provided in Table 6.

### 4.4. Performance Benchmarking

#### 4.4.1. Physical Interpretation of Sample-to-Sample Variability

The observed sensitivity difference between Sample 1 and Sample 2 exceeds the run-to-run scatter within each fixed sample and wiring configuration, indicating that design- and microfabrication-induced variation, together with connection configuration, is the main source of dispersion rather than random electrical noise alone. Under identical test protocols, the mean effective Seebeck coefficient differs by approximately 1.1–1.2 μV/°C between the two samples, corresponding to a relative difference of ~2.7–3.0%. The small run-to-run variation under each fixed configuration suggests that the tested devices and acquisition setup are repeatable over the primary calibration range. For industrial deployment, this result supports batch-level calibration verification rather than assuming a universal sensitivity for all fabricated sensors.

This variability is closely related to the differentiated device designs. Sample 1 employs a ZnO dielectric layer, a NiCr/Au thermoelectric pair, and a rectangular hot junction. It is included as a comparative NiCr-based structure for flexible, high-signal-to-noise-ratio sensing and for examining the measurement-chain-aware calibration strategy on a second NiCr-based material pair, rather than as a replacement for the NiCr/NiSi system. Au was selected because of its high electrical conductivity and chemical stability within the tested temperature range. Sample 2 employs an Al_2_O_3_ dielectric layer, a NiCr/NiSi thermoelectric pair, and a different rectangular hot-junction geometry, and is optimised for industrial high-temperature cutting measurement. The calibrated effective coefficient is governed not only by the Seebeck coefficient difference between the two thin-film legs, but also by the layer stack, thermal-boundary condition, connection route, and reference condition. Pad-to-wire transitions may also introduce dissimilar-metal junctions, which can generate parasitic thermoelectric voltages when local temperature gradients are present [11,21].

In magnetron-sputtered thin films, thermoelectric properties can be influenced by grain size, crystallographic texture, film density, residual stress, compositional uniformity, oxidation state, and interfacial quality [1,11]. This interpretation is consistent with reported thickness-dependent Seebeck coefficient variation in NiCr/NiSi films [11] and crystallographic-orientation-dependent precision in highly textured Pt/Ir films [18]. Because these microstructural parameters were not directly measured in the present study, the mechanism discussion is limited to physically plausible interpretations supported by the calibration data and device design information. Overall, the results show that TFTC sensitivity depends on the combined effects of thermoelectric material pair, device structure, electrical-contact condition, and thermal-boundary condition. Therefore, the calibration record should include the wiring configuration and reference condition together with the calibration coefficients and uncertainty budget. Systematic microstructural characterisation remains necessary for identifying the root causes of performance variation and establishing process-control tolerances [7].

#### 4.4.2. Measurement Uncertainty Budget and Digital-Twin-Ready Data Package

A standardised uncertainty budget was prepared in accordance with the Guide to the Expression of Uncertainty in Measurement (GUM). It combines Type A uncertainty from measurement repeatability with Type B contributions from instrument specifications, as detailed in Table 7. The budget was evaluated at 260 °C with a coverage factor of k = 2, corresponding to a 95% confidence level. The evaluated terms include slope repeatability, reference temperature, cold-junction compensation, voltage measurement/resolution, and regression residuals. Yang proposed an integrated calibration-coefficient approach for thin-film heat flux gauges and achieved a relative expanded uncertainty below 7% [24]. In the present TFTC study, the calibration record focuses on the tested wiring configuration and the uncertainty terms directly included in the calibration setup.

Uncertainty propagation follows the inverse calibration model used to reconstruct temperature from the measured thermoelectric potential *E*. Slope repeatability and model residuals act through the fitted voltage–temperature relation, while reference-temperature, cold-junction compensation, and voltage-resolution terms enter as additive or equivalent temperature uncertainties. The relative contribution of each term can be estimated using ui^2^/uc^2^, where ui is an individual standard uncertainty component and uc is the combined standard uncertainty. The slope-related contribution increases with the temperature difference from the reference calibration temperature, whereas reference-temperature and voltage-resolution terms remain closer to constant over the primary range. Outside the calibrated range, nonlinear Seebeck behaviour may introduce additional error if a first-order model is used without higher-order correction. Fabrication variability may also affect uncertainty through the sample-specific effective Seebeck coefficient, contact condition, pad-to-wire transition, and layer-stack thermal-boundary condition. Because only two independently designed TFTC samples were examined, this effect is discussed qualitatively rather than treated as a statistically quantified uncertainty term.

To support future digital integration, the calibration data packet is defined as a compact calibration record associated with each calibrated TFTC sample and wiring configuration. It contains the calibrated TFTC sample, wiring configuration, calibration model and coefficients, validity range, and GUM-compliant uncertainty budget (Table 8). This structure links the calibration coefficients to the tested wiring configuration and reference condition and supports traceable digital interpretation of TFTC temperature data [23,35].

The present uncertainty budget applies to the tested calibration configuration and to the uncertainty terms directly evaluated in the laboratory setup. Deployment-dependent effects, including parasitic thermoelectric voltages at connector contacts outside the tested interface, signal-conditioning electronics, non-isothermal field wiring, and environmental electromagnetic interference, were not quantitatively evaluated in this budget. These effects should be considered when the wiring route or electromagnetic environment differs from the calibration setup. Connector isothermalisation, shielding, grounding, and configuration-specific recalibration can help maintain consistency between calibration and deployment conditions.

**Figure 7:**
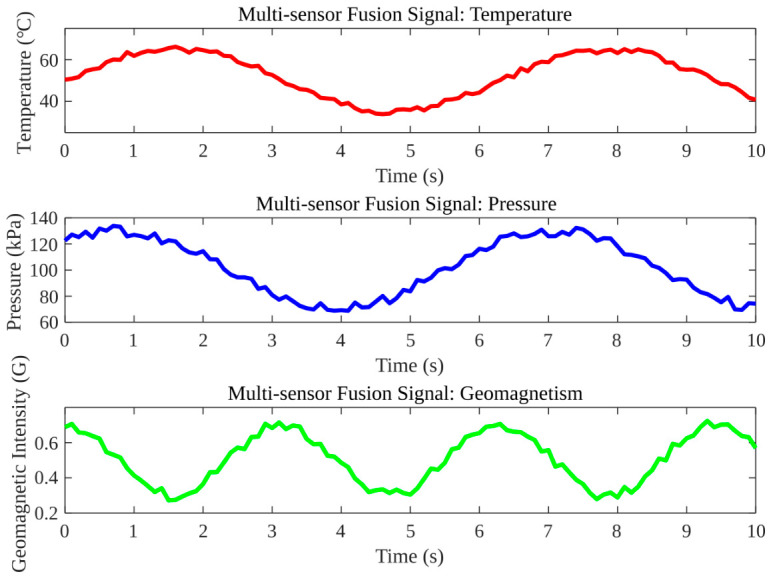
Illustrative simulated multi-sensor fusion signals in a digital twin framework.

The calibration data packet is intended to preserve this configuration dependence and reduce the risk of calibration misuse. A TFTC calibrated with copper leads should not be used with compensation wires unless recalibration or a validated correction is performed. By storing the wiring configuration, validity range, uncertainty budget, and calibration coefficients together, the packet allows future digital monitoring systems to determine whether incoming temperature data are being interpreted within the tested calibration configuration. Figure 7 provides an illustrative multi-sensor fusion waveform to show how the calibrated TFTC temperature channel may be combined with other sensing modalities for system-state interpretation in a future digital twin framework. The figure is intended as an application-oriented illustration of the calibration data packet concept rather than a completed system-level digital twin validation.

#### 4.4.3. Research Limitations and Industrial Deployment Implications

The present study is a calibration-focused evaluation of two NiCr-based TFTC configurations under specified laboratory conditions. It covers static calibration, wiring-configuration effects, the uncertainty terms included in the tested setup, short-term high-temperature verification, and apparent transient response. Therefore, the results should not be interpreted as complete validation of industrial lifetime, intrinsic junction bandwidth, or material-level structure–property relationships. Material characterisation was limited to process-defined layer information and optical inspection. The reported Seebeck coefficient is therefore a sensor-level effective coefficient obtained from the tested TFTC calibration configuration under a specified wiring condition, not the intrinsic Seebeck coefficient of an individual NiCr, NiSi, or Au thin-film. Quantitative film conductivity, absolute Seebeck coefficient, and microstructure–performance correlation require dedicated single-leg test structures and crystallographic, compositional, interfacial, stress-related, and high-temperature-evolution analyses. The uncertainty budget applies to the sensing unit and its immediate tested wiring interface. Parasitic thermoelectric potentials outside this interface, signal-conditioning electronics, non-isothermal field wiring, and environmental electromagnetic interference were not quantitatively evaluated. The 1000 °C test provides short-term extended-range verification for Sample 2, while long-term drift, oxidation, thermal cycling, mechanical loading, and interface degradation require dedicated reliability testing. The dynamic metrics describe an apparent system-level transient response; intrinsic junction response, thermal-contact effects, substrate/protective-layer heat-conduction delay, and frequency-domain bandwidth require controlled transient or frequency-response analysis [16,17]. Despite these limits, the present work provides a structured calibration workflow and traceable uncertainty report for the tested flexible NiCr-based TFTC configurations. The calibration data packet records device identity, wiring configuration, calibration coefficients, validity range, and uncertainty information, supporting consistent interpretation of TFTC data in future digitally integrated monitoring and manufacturing-oriented digital-twin workflows [23,36].

#### 4.4.4. Practical Installation, Protection, and Signal-Transfer Considerations for Milling Applications

**Figure 8:**
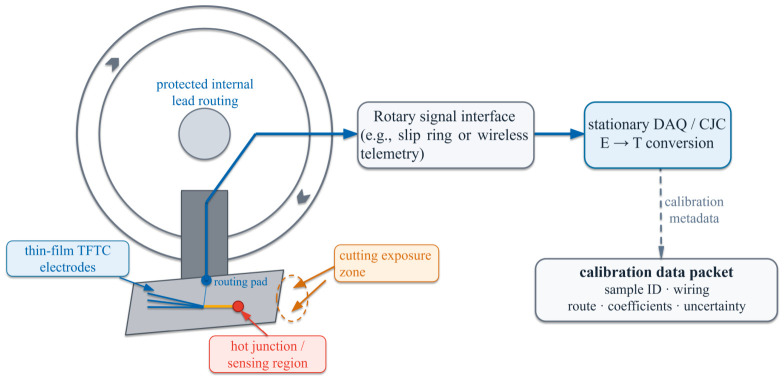
Conceptual schematic of a representative rotating-tool signal-transfer route for TFTC-based milling temperature monitoring. The TFTC sensing region is schematically positioned on the insert region near the tool-workpiece thermal zone, and the thin-film electrodes are connected to a routing pad and protected lead path. The thermoelectric potential *E* can be transferred to a stationary DAQ/CJC unit through a rotary signal interface, such as a slip ring or wireless telemetry module, and interpreted using the calibration data packet. In the schematic, the blue solid arrows indicate the thermoelectric signal-transfer path, the grey circular arrows indicate tool rotation, the grey dashed arrow indicates calibration-metadata use for data interpretation, and the orange arrows indicate the cutting-exposure zone. The schematic is not to scale. The schematic is not to scale.

For milling-related temperature monitoring, a practical installation position for a TFTC is close to the tool-workpiece thermal zone while avoiding the most severe direct mechanical contact at the cutting edge. Figure 8 schematically illustrates a representative integration route. In this concept, the thin-film sensing region is placed on the insert region near the local thermal source, the electrode traces are connected to a routing pad, and the signal path is routed through a protected internal lead region toward a rotary signal interface.

Protection is an essential consideration because chips, coolant, and mechanical contact may damage exposed thin films, electrode pads, and lead transitions. In the present device design, a SiO_2_ protective overcoat was deposited over the device surface except for the electrode pads to mitigate oxidation and environmental degradation during testing. For machining-oriented deployment, this protection concept may be combined with local encapsulation, recessed or shielded lead routing, strain-relieved electrical connections, and placement of the routing pad away from the primary chip-flow and coolant-impact region. For rotating milling tools, the measured thermoelectric potential *E* can be transferred from the rotating tool side to stationary acquisition hardware through a rotary signal interface, such as a slip ring or wireless telemetry module, followed by a stationary data-acquisition and cold-junction- compensation unit.

Because the wiring route, connector temperature, and reference condition can affect the effective calibration, the selected signal-transfer route should be treated as part of the calibrated measurement chain. The calibration data packet therefore preserves the calibrated TFTC sample, wiring route, calibration coefficients, validity range, and uncertainty information so that voltage-to-temperature conversion is applied under the same measurement-chain condition used for calibration, or under a configuration for which a validated correction has been established. Figure 8 is intended to clarify the installation and signal-transfer concept for milling applications; it is a conceptual schematic and is not drawn to scale.

## 5. Conclusions and Outlooks

### 5.1. Key Conclusions

This study presents a wiring-configuration-aware calibration approach for flexible NiCr-based thin-film thermocouples under specified laboratory conditions. Two TFTC variants, NiCr/Au and NiCr/NiSi, were fabricated on flexible polyimide substrates and characterised over 20–260 °C, with short-term extended verification up to 1000 °C for the NiCr/NiSi sample. The NiCr/NiSi sample is the principal type-K-equivalent configuration in this work, whereas the NiCr/Au sample serves as a comparative NiCr-based structure for evaluating the calibration approach on a second material pair. Temperature reconstruction against a traceable type-K standard reference yielded a maximum absolute deviation of 0.27 °C, a root-mean-square error of 0.15 °C, and a relative error within ±0.5% over the primary calibration range. Repeated calibration runs showed CV < 0.3%, indicating good repeatability under each fixed sample and wiring configuration. The comparison between copper-lead and compensation-wire configurations showed a sensitivity shift of approximately 0.40–0.44 μV/°C, corresponding to a relative change of ~1%. Therefore, a TFTC calibrated with one wiring scheme should be used under the same wiring configuration unless recalibration or a validated correction model is applied. The proposed calibration data packet records the calibrated TFTC sample, wiring configuration, calibration model and coefficients, validity range, and GUM-compliant uncertainty budget, supporting consistent interpretation of the tested calibration configurations in future digital monitoring systems. The 1000 °C result demonstrates short-term thermoelectric signal generation under the present laboratory conditions, while the reported dynamic metrics describe apparent system-level transient behaviour rather than intrinsic junction bandwidth.

### 5.2. Future Outlooks

Future work will extend this calibration-focused study toward material-level characterisation, long-term reliability, intrinsic dynamic analysis, deployment-side uncertainty evaluation, milling-oriented implementation, and practical digital integration. Dedicated single-leg NiCr, NiSi, and relevant counter-electrode test structures will be prepared to measure film electrical conductivity and material-level Seebeck coefficients under controlled conditions [11]. Crystallographic, compositional, interfacial, stress-related, and oxidation-related analyses will be used to clarify the relationship between film quality and TFTC sensitivity and stability [11,32]. Reliability will be assessed through sustained high-temperature exposure, thermal cycling, drift evaluation, and application-relevant mechanical and environmental loading [27,32]. Oxidation- and wear-resistant encapsulation layers, including Mg-doped Al_2_O_3_-based coatings, will also be considered for improved environmental robustness [27]. For milling-related deployment, protected tool-insert mounting and rotating-to-stationary signal transfer will be evaluated under representative machining environments [2,15]. Deployment-side parasitic thermoelectric effects, signal-conditioning influences, non-isothermal field wiring, and environmental electromagnetic interference will be examined under defined wiring and acquisition conditions. Controlled transient excitation and frequency-response methods will be used to separate junction response, thermal-contact effects, substrate/protective-layer heat-conduction delay, and measurement-system bandwidth [16,17]. The calibration data packet will also be evaluated in a practical digital monitoring workflow to examine how calibration metadata and uncertainty information support multi-sensor temperature-data interpretation [23,35].

## Figures and Tables

**Table 1:** Practical trade-offs among representative thermoelectric and electrode materials relevant to TFTC sensing.

Material	Advantages	Limitations	Typical Role
Bi_2_Te_3_-based compounds	High thermoelectric performance near room temperature; mature thermoelectric energy-conversion material family	Not generally selected as a robust thermocouple electrode for high-temperature TFTCs; stability and processing requirements differ from Ni-based thermocouple alloys	Background thermoelectric material for comparison; not the electrode material used in this work
Ag	High electrical conductivity; low resistivity; easy patterning	Poor oxidation resistance at high temperature; migration risk	Low-temperature electrode/interconnect
Ni	Low cost; good compatibility with Ni-based thermoelectrodes; moderate oxidation resistance with coatings	Oxidises at elevated temperature without protection; potential diffusion	Thermocouple leg/interconnect
Pt	Excellent chemical and oxidation stability; suitable for harsh/high-temperature environments	High cost; lower Seebeck coefficient than some alloys; requires adhesion layers on some substrates	High-temperature thermocouple/reference electrode
Ti	Good adhesion to many substrates; forms stable oxides; can serve as an adhesion layer	Higher resistivity than Ag/Cu; oxidation changes properties	Adhesion/transition layer

**Table 2:** Static calibration data and linearity error of the tested TFTC calibration configuration (Sample 2, compensation-wire configuration, average of five repeated runs).

Standard T (°C)	Measured Thermoelectric Potential, *E* (mV)	Reconstructed Temperature (°C)	Error (%)	Abs. Error (°C)
20.0	0.812	19.904	−0.48	0.096
50.0	2.025	50.117	0.23	0.117
80.0	3.24	80.102	0.13	0.102
110.0	4.455	110.266	0.24	0.266
140.0	5.67	140.023	0.02	0.023
170.0	6.885	169.828	−0.1	0.172
200.0	8.1	200.121	0.06	0.121
230.0	9.315	230.008	0.0	0.008
260.0	10.53	259.925	−0.03	0.075

**Table 3:** Seebeck coefficient (effective sensitivity) statistics derived from five repeated static calibration runs per condition (20–260 °C).

Condition	Seebeck, *S_eff_* (µV/°C), Mean ± SD	SD (µV/°C)	CV (%)	Min	Max	*n*
Sample 1, copper leads	41.646 ± 0.052	0.052	0.13	41.588	41.717	5
Sample 2, copper leads	40.527 ± 0.114	0.114	0.28	40.423	40.720	5
Sample 1, compensation wires	41.244 ± 0.074	0.074	0.18	41.164	41.337	5
Sample 2, compensation wires	40.092 ± 0.056	0.056	0.14	40.056	40.187	5

**Table 4:** Cycle repeatability test results of the fabricated TFTC (fixed test temperature: 100 °C).

Test Cycle	Measured Temperature (°C)	Absolute Deviation from Initial Calibration Value (°C)
1	100.0951	0.0951
5	100.0254	0.0254
10	99.7155	0.2845
15	99.7121	0.2879
20	100.0429	0.0429

**Table 5:** Seebeck coefficient comparison of representative thermocouple types (20–260 °C).

Thermocouple Type	Effective Seebeck Coefficient (μV/°C)
Fabricated NiCr/NiSi TFTC (This work)	40.09–41.65
Standard K-type wire thermocouple	~41.2
Standard J-type wire thermocouple	55.0

**Table 6:** Comparative summary of representative TFTC studies (2022–2025) across key performance dimensions evaluated in this work.

Material System	Seebeck (μV/°C)	Temp. Range (°C)	Max. Error/Repeatability	Wiring Config. Quantified	Ref.
NiCr/NiSi TFTC (carbide)	48.13	up to 600	—	No	[2]
NiCr/NiSi TFTC (PI)	38.9–39.1	20–350	CV < 0.3%	No	[3]
NiCr/NiSi TFTC (alumina)	40.72	Milling	R^2^ = 96.76%	No	[15]
NiCr/NiSi TFTC (Al alloy)	40.69	30–180	—	No	[25]
NiCr/NiSi TFTC (tool)	34.96–34.97	Cutting	Error < 5%	No	[34]
Pt-Rh10/Pt TFTC (alumina)	~10.70	50–1500	Repeat. err. 0.08%	No	[8]
Pt/Ir TFTC	—	300–1000	Deviation < 0.21%	No	[18]
ITO/In_2_O_3_ TFTC (alumina)	129.3	200–1450	Error ~7.69%	No	[20]
This work	40.09–41.65	20–260	Maximum absolute error ≤ 0.27 °C; CV < 0.3%	Yes	none

Note: ‘—’ indicates that the metric was not explicitly reported in the cited study. Direct numerical comparison across studies with different temperature ranges, substrates, and test protocols should be interpreted with caution. All data values are as reported in the respective references, verified via DOI-based retrieval where accessible.

**Table 7:** Measurement uncertainty budget for the TFTC measurement chain (evaluated at 260 °C, coverage factor k = 2, 95% confidence level).

	Source	Type	Contribution	Comment
Standard Uncertainty Components	Slope repeatability (u_a_)	A	0.432 °C	Converted from the five-run Seebeck coefficient SD; evaluated between 20 °C and 260 °C
	Reference temperature (u_ref_)	B	0.05 °C	1σ standard uncertainty of the standard K-type reference thermocouple
	Cold-junction compensation (u_CJC_)	B	0.10 °C	Standard uncertainty associated with cold-junction compensation of the thermocouple input module
	Voltage measurement/resolution (u_V_)	B	0.05 °C	Equivalent temperature uncertainty from data acquisition system voltage resolution
	Model fit/residuals (u_fit_)	B	0.05 °C	1σ standard uncertainty from linear regression residuals of the calibration curve
Calculated Uncertainty Results	Combined standard uncertainty (u_c_)		0.452 °C	Calculated via root-sum-square (RSS) of all independent uncertainty components
	Expanded uncertainty (U, k = 2)		0.904 °C	*U* = *k* × *u_c_* Corresponding to ~95% confidence level

**Table 8:** Proposed calibration data packet for future digitally integrated TFTC monitoring systems.

Packet Item	Content Defined in This Work	Function in Digital Integration
Calibrated TFTC sample	Sample 1 (NiCr/Au) or Sample 2 (NiCr/NiSi)	Links the calibration record to the specific TFTC sample to avoid applying coefficients to the wrong device.
Wiring configuration	Copper-lead configuration or compensation-wire configuration	Ensures that calibration coefficients are used only with the wiring topology under which they were obtained.
Calibration model and coefficients	First-order linear calibration model; effective Seebeck coefficient a; offset b; reference calibration temperature T0; inverse temperature reconstruction model	Converts the measured thermoelectric voltage into reconstructed temperature.
Validity range	20–260 °C for the primary static calibration; extended verification up to 1000 °C for Sample 2	Prevents the calibration model from being applied outside the tested temperature range.
Uncertainty budget	Type A contribution from slope repeatability; Type B contributions from the reference thermocouple, cold-junction compensation, voltage measurement/resolution, and model residuals; combined standard uncertainty; expanded uncertainty at k = 2	Provides a traceable uncertainty statement for future digital monitoring and multi-sensor data interpretation.

## Data Availability

The data used to support the findings of this study are available from the first author upon reasonable request.

## Supplementary Materials

The following supporting information can be downloaded at: https://www.mdpi.com/article/10.3390/mi17060746/s1, Figure S1: Histogram of absolute temperature reconstruction error over the 20–260 °C calibration range; Figure S2: Temperature reconstruction error map versus temperature and experiment index; Figure S3: Comparison of reconstructed temperature from the TFTC calibration model and standard reference temperature; Figure S4: Dynamic response waveform of the TFTC under step-temperature excitation; Figure S5: Extended-range calibration curve up to 1000 °C and linear fit; Table S1: Procedural fabrication and packaging details supporting the fabrication description.

## Abbreviations

The following abbreviations are used in this manuscript:

TFTC	Thin-film thermocouple
CV	Coefficient of Variation
EMF(E)	Electromotive Force
S	Seebeck coefficient
CJC	Cold-junction compensation
RMSE	Root-mean-square error
