# Supplementary material for: Static Calibration and Wiring-Configuration-Dependent Performance of NiCr-Based Thin-Film Thermocouples
Source: Micromachines (Basel). 2026 Jun 20;17(6):746. doi: 10.3390/mi17060746 (PMC13302832; doi:10.3390/mi17060746)
Supplement: Supplementary file 1 [file micromachines-17-00746-s001.zip › Kang_Yuan_SupplementaryMaterials_20260620.pdf]

Supplementary Materials

This document contains five supplementary figures. Figure S1 and Figure S2 provide diagnostic visualisation of the temperature reconstruction error. Figure S3 presents the full-resolution dataset underlying the representative curves in the main text. Figure S4 offers a complementary view of the dynamic response. Figure S5 presents an optimised subset of the high-temperature calibration data. Table S1 provides the procedural fabrication and packaging details for substrate preparation, patterning, electrode-pad opening, wire bonding, curing, and lead protection, which were moved from the main text to improve readability while preserving process traceability.

Figure S1. Histogram of absolute temperature reconstruction error over the 20–260 °C calibration range. The histogram pools pointwise errors $|T_{TFTC} - T_{ref}|$ from 25 data points. Summary statistics derived from this distribution.

Figure S2. Temperature reconstruction error map versus standard reference temperature and experimental run index. Each cell represents the absolute error $|T_{TFTC} - T_{ref}|$ for a single calibration run at a given temperature setpoint, with colour intensity indicating error magnitude (see colour bar). The absence of systematic colour gradients along either axis confirms that the linear calibration model does not exhibit temperature-dependent drift or progressive degradation across repeated runs.

Figure S3. Comparison of reconstructed TFTC temperature and standard reference temperature. The main-text figure displays the nine standardised calibration setpoints (20, 50, 80, 110, 140, 170, 220, 230, and 260 °C). This figure presents the full dataset acquired at 10 °C intervals over the 20–260 °C range, confirming that the linear reconstruction behaviour observed at the nine representative setpoints is characteristic of the entire calibration span.

Figure S4. Dynamic response waveform of the TFTC under step-temperature excitation. The main-text figure presents the thermoelectric voltage output (mV) versus temperature (°C). This figure presents the same transient event as reconstructed temperature (°C) versus time (ms), providing a direct visualisation of the temporal evolution of the sensed temperature. The dynamic metrics extracted from this waveform (10%–90% rise time: ~56 ms; time-to-peak: ~95 ms; settling time: 120 ms) are reported in the main text and compiled in Table 5.

Figure S5. Extended-range calibration curve up to 1000 °C with linear fit. The main-text figure displays all test points acquired during the high-temperature calibration campaign. This figure presents an optimised subset of 11 selected data points to illustrate the fidelity of the linear approximation. The fitted effective Seebeck coefficient (39.79 μV/°C) and correlation coefficient ($R^2 > 0.99$) are consistent with the values reported in Section 4.1.3.

Table S1. It summarises the procedural fabrication and packaging details that were moved from the main text to improve readability while preserving process traceability. The main manuscript retains the key device design and deposition parameters, including the material pairs, layer stacks, nominal thicknesses, hot-junction dimensions, sputtering pressure, target-to-substrate distance, sputtering power density, target purity, target compositions, and protective-layer design. The supplementary table provides the corresponding implementation details for substrate preparation, patterning, electrode-pad opening, wire bonding, curing, and lead protection.

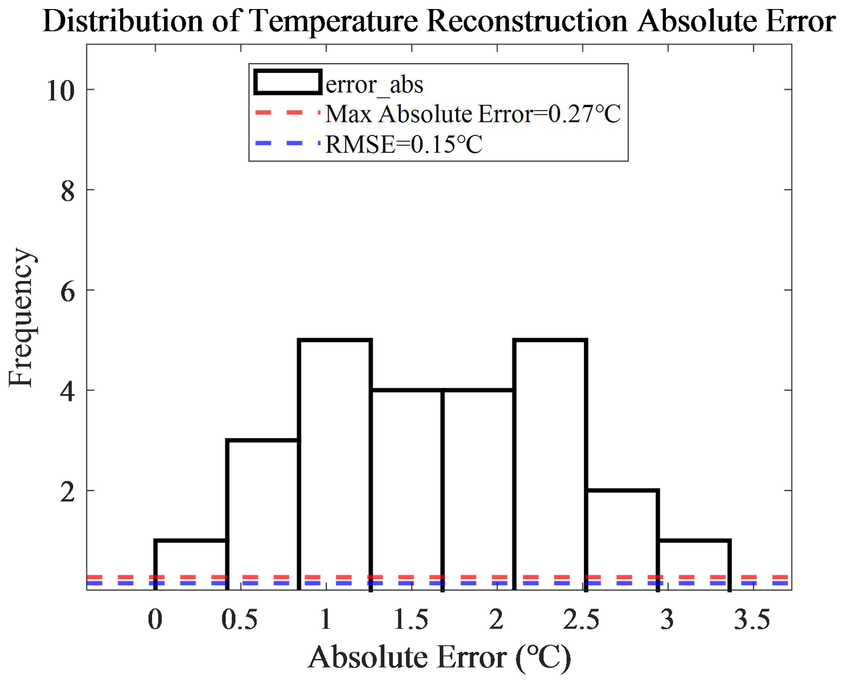

Figure S1. Histogram of absolute temperature reconstruction error over the 20–260 °C calibration range.

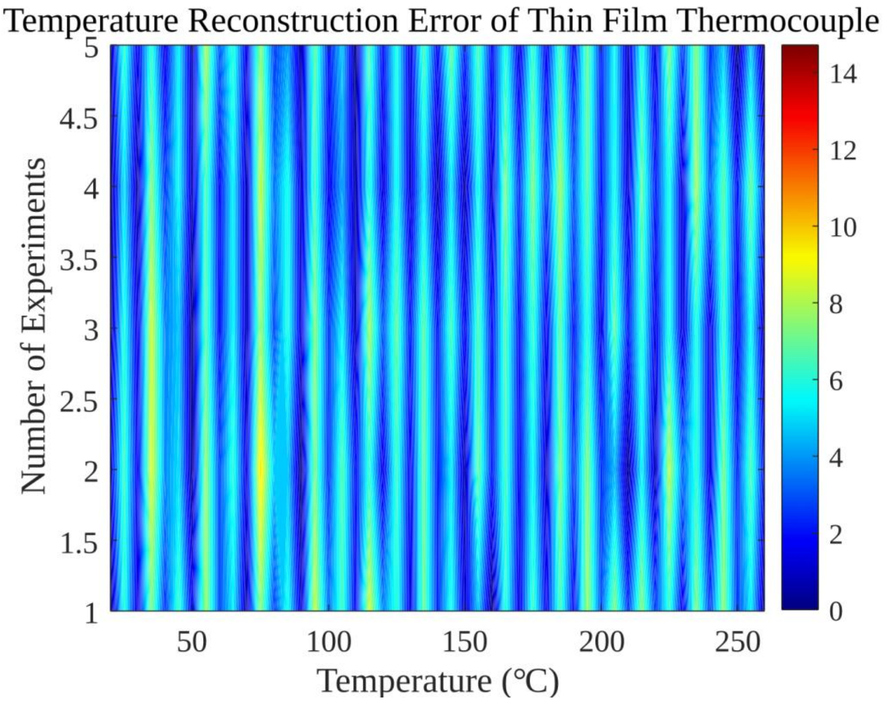

Figure S2. Temperature reconstruction error map versus temperature and experiment index (color values as shown).

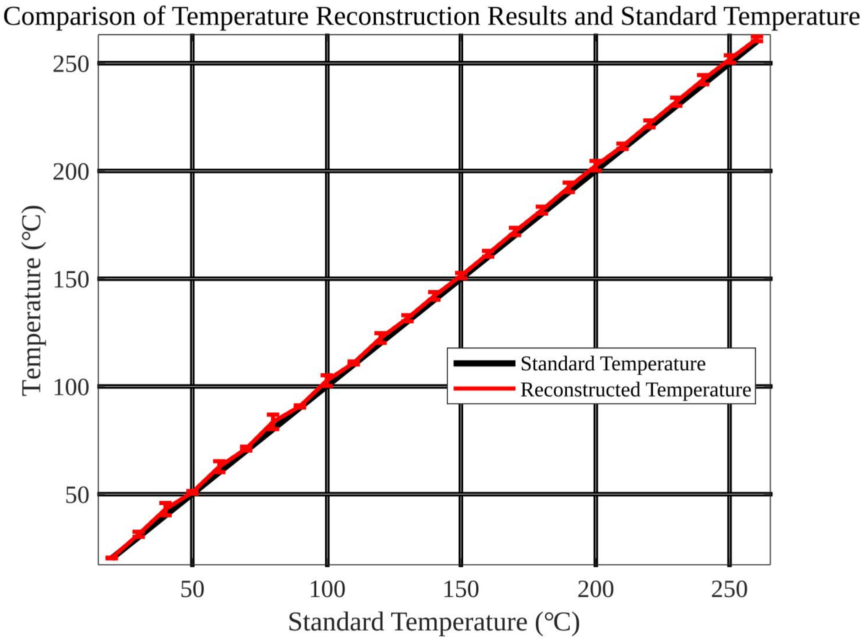

Figure S3. Comparison of reconstructed temperature from the TFTC calibration model and standard reference temperature.

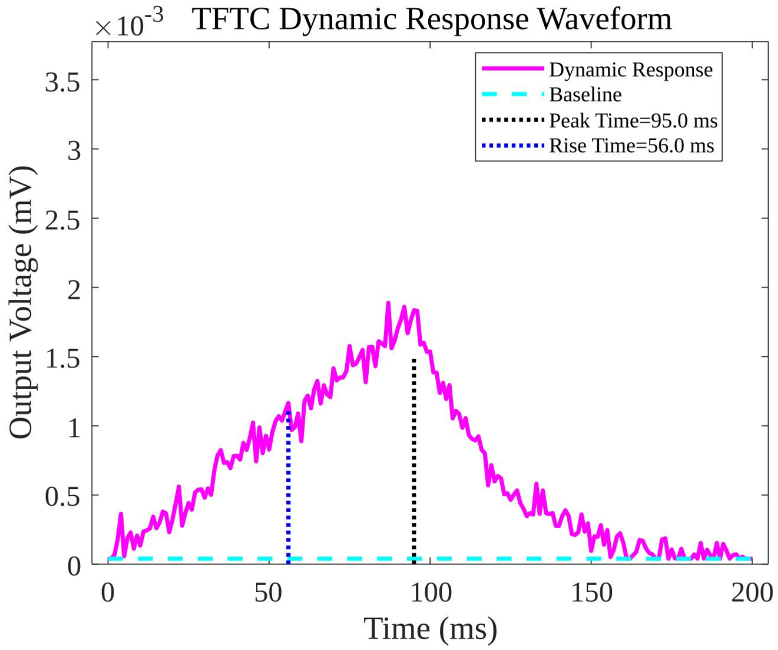

Figure S4. Dynamic response waveform of the TFTC under a step temperature excitation.

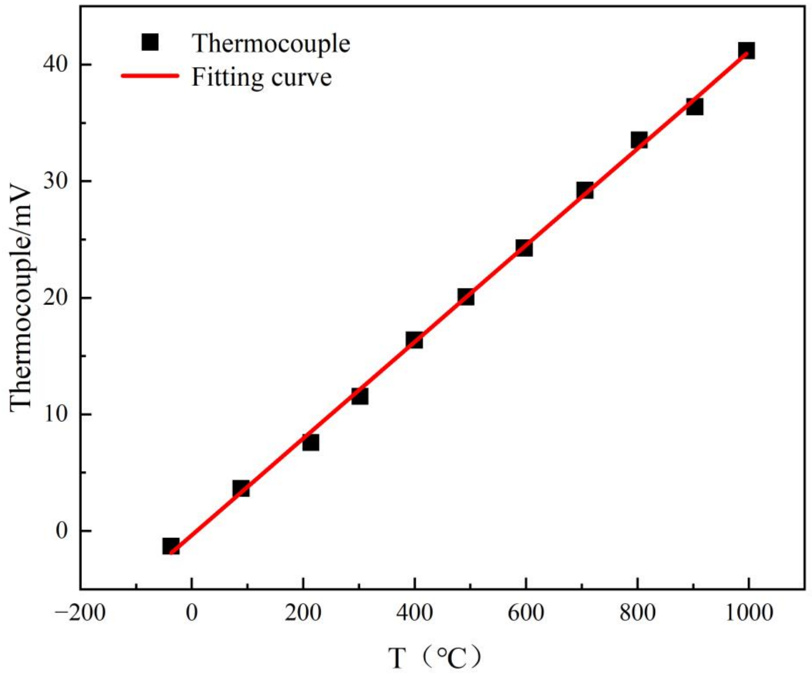

Figure S5. Extended-range calibration curve up to 1000 °C and linear fit.

Table S1. Procedural fabrication and packaging details supporting the fabrication description

Process item	Detail explicitly reported in the manuscript
Substrate cleaning	Sequential ultrasonic cleaning in organic solvents and deionised water
Substrate drying	Nitrogen blow-drying after cleaning
Moisture removal	Low-temperature baking before deposition
Patterning implementation	Thermoelectric films were patterned using customised metal shadow masks
Protective-layer opening	The SiO_2 protective overcoat was deposited over the device surface except the electrode pads
External wire bonding	External measurement wires were bonded to the electrode pads using high-temperature conductive silver paste
Curing condition	The conductive silver paste was cured at room temperature
Lead protection	Wire leads were strain-relieved to prevent mechanical damage during handling
